# Prevalence of *Helicobacter pylori vacA*, *cagA*, *iceA *and *oipA *genotypes in Tunisian patients

**DOI:** 10.1186/1476-0711-9-10

**Published:** 2010-03-19

**Authors:** Khansa Ben Mansour, Chédlia Fendri, Meriem Zribi, Afef Masmoudi, Mounir Labbene, Azza Fillali, Nabil Ben Mami, Taoufik Najjar, Ahmed Meherzi, Tahar Sfar, Christophe Burucoa

**Affiliations:** 1 Microbiology laboratory/UR04SP08 Rabta University Hospital, Tunis, Tunisia; 2 Gastroenterology unit, Menzel Bourguiba Hospital, Tunisia; 3 Gastroenterology A unit, Rabta University Hospital, Tunis, Tunisia; 4 Gastroenterology B unit, Rabta University Hospital, Tunis, Tunisia; 5 Gastroenterology unit, Charles Nicolle University Hospital, Tunis, Tunisia; 6 Pediatric Unit Mongi Slim University Hospital, La Marsa, Tunis, Tunisia; 7 Pediatric Unit Tahar Sfar Hospital, Mahdia, Tunisia; 8 Université de Poitiers, EA 3807, Poitiers 86021, France; Laboratoire de bactériologie, Poitiers 86021, France

## Abstract

**Background:**

Distinct virulence factors of *H. pylori* have been described: the vaculating cytotoxin (vacA), the cytotoxin associated gene (cagA), the induced by contact with epithelium factor Antigen (iceA gene) and the outer membrane protein oipA. In Tunisia, there are no data regarding the pattern of H. pylori genotypes; therefore, this prospective and multicentre study was the first to be done in Tunisia and aimed to investigate the prevalence of the vacA, cagA, iceA and oipA genotypes of *H. pylori* isolates from Tunisian patients with peptic ulceration, gastric cancer, MALT lymphoma and gastritis.

**Methods:**

*H. pylori* was cultured from endoscopic biopsies obtained from 281 Tunisian patients. The vacA alleles, cagA, iceA and oipA genotypes were determined by PCR.

**Results:**

The vacA s1m1, s1m2 and s2m2 were respectively found in 10.7%, 12.5% and 45.6% of strains. The s2m1 genotype was not detected in our study. The cagA was found in 61.6% of isolates. The iceA1 and the iceA2 genotypes were respectively isolated in 60.2% and in 16% of strains. The oipA genotype was detected in 90.8% of strains. Considering the vacA and iceA genotypes, the presence of multiple *H. pylori* strains in a single biopsy specimen was found respectively in 31.4% and 23.8%. The comparison between strains isolated from antrum and fundus showed that Tunisian patients were infected with two or more strains of different cagA, vacA, iceA and oipA genotypes and the discordance was respectively in 9.6%, 4.6%, 8.9% and 8.5% of strains.

**Conclusion:**

Our results showed that in 46% (131 strains among 281), the *H. pylori* strains were highly virulent in relation of the three or four virulent factors they could carry. These finding were described before in the literature. Tunisian patients were colonized by one or multiple strains of *H. pylori *in the same time in relation of presence of vacA m1/m2 and iceA1/iceA2 in the same biopsy. The discordance between strains isolated from antrum and fundus was high, and it is in favour of multicolonization.

## Background

*Helicobacter pylori *(*H. pylori*) is one of the most common bacterial pathogens of humans and has a worldwide distribution. Infection by *H. pylori *is associated with the development of chronic gastritis, gastric or duodenal ulcer, gastric cancer and MALT-lymphoma [1]. Different virulence genes have been described in *H. pylori *infection such as *cagA*, *vacA*, *iceA *and *oipA *genes. The cytotoxin-associated gene (*cagA*) is frequently associated with cytotoxin production and the induction of interleukin 8 (IL8) by gastric epithelial cells [2]. Several studies have suggested that *cagA *is a useful marker for the most virulent strains that are associated with peptic ulcer, atrophic gastritis and adenocarcinoma [3-5]. The cag pathogenicity island (PaI) encodes a type IV secretory system and delivers CagA into the host cytosol where becomes phosphorylated on tyrosine residue. Phosphorylated CagA interacts with the phosphatase SHP-2 causing dephosphorylation of cortactin and cytoskeletal rearrangements forming the "hummingbird" phenotype [6]. The vacuolating cytotoxin gene (*vacA*) is an important virulence factor encoding the vacuolating toxin and it is present in all strains [7,8]. There is a considerable variation in vacuolating activities among strains [9-11] due to the sequence heterogeneity at the middle (m) and the signal (s) regions within the *vacA *gene. The middle region has m1 and m2 allelic types and the signal region located at the 5' end of the gene has an s1 (s1a, s1b and s1c) or an s2 allele. Strains carrying the s1ml mosaic combination of the gene *vacA *exhibit higher levels of cytotoxic activity than s1m2 strains, while s2m2 strains do not secrete the vacuolating cytotoxin [12]. Another virulence gene designated *iceA *(for induced by contact with epithelium) has been recently described. The *iceA *gene has two main allelic variants *iceA*1 and *iceA*2 [13] but the function of these variants is not yet clear [14-18]. *iceA*1 is upregulated upon contact of *H. pylori *with the gastric epithelium and has been regarded as a marker for peptic ulcer disease [19]. Recently, a novel putative virulence factor has been identified, the *oipA *(outer inflammatory protein) gene, which encodes one of the outer membrane proteins and is an inflammation-related gene located approximately 100 kb from the *cag *PAI on the *H. pylori *chromosome [20]. *oipA *induces IL-8 secretion by epithelial cells and active OipA protein production may be "on" or "off" depending on the number of CT dinucleotide repeats in the signal sequence of the *oip*A gene (HP0638) [21].

In Tunisia, there are no data regarding the pattern of *H. pylori *genotypes in patients; therefore, our prospective and multicentre study was the first to be done in Tunisia and to our knowledge the first one in North Africa. It aimed to investigate the prevalence of the *vacA*, *cagA*, *iceA *and *oipA *genotypes of *H. pylori *isolates from Tunisian patients.

## Materials and methods

### Materials

Biopsy samples were obtained over a 2 years-period (March 2005 to August 2007) from patients referred for endoscopy at 6 centers of gastroenterology in Tunisia. 281 patients, who had *H. pylori *positive cultures, were enrolled in this study. The mean age of the patients was 39.9 (range, 2 to 88) and 54.4% were women. Regarding macroscopic aspect of the mucosa and histological routine results, patients were distributed into gastritis in 195 cases (69.3%), peptic ulceration in 78 cases (27.7%) (duodenal ulcer in 67 cases, gastric ulcer in 11 cases), MALT lymphoma of the stomach in one case (0.3%) and gastric cancer in 7 cases (2.5%). One endoscopic biopsy specimen taken from the antrum and one taken from the fundus were immediately frozen and kept separately at -80°C. In addition, one biopsy specimen from the antrum and one from the fundus were used for the culture. In our study, we also obtained colonies isolated separately from two biopsy sites (antrum and fundus) to determine if one patient can be colonized with one or more strains of *H. pylori*. All subjects were answering to questionnaire related to age, sex, gastric or duodenal peptic ulcer diseases upon endoscopy.

#### Culturing of *H. pylori *and DNA extraction

The biopsy specimens were cut into small pieces, homogenized in a Petri dish with a sterile scalpel and were smeared on the surface of Columbia agar plates containing 10% horse blood and Skirrow (Oxoid, England) supplement. Incubation was performed in microaerophilic conditions at 37°C for a maximum of 6 days. The *H. pylori *colonies were smooth, translucent and small (2 mm of diameter). In our study, the antral and fundic biopsy specimens were studied separately. Colonies that exhibited the described characteristic morphologies were identified as *H. pylori *if they were Gram negative and shaped bacilli, and urease, catalase and oxidase positive. From the primary growth, seven or eight colonies were pooled together, and genomic DNA was extracted with the QIAamp DNA mini kit (Qiagen, Germany) according to the manufacturer's instructions. The isolated DNA was eluted in 200 μl of 1× TE buffer (10 mM Tris-HCl, 1 mM EDTA [pH 8.0]) and stored at -20°C until use.

### *H. pylori *genotyping

After DNA extraction, polymerase chain reactions (PCR) were performed in a volume of 50 μl containing 1 μM of each primers, 1 μL of genomic DNA (approximately 200 ng), 1 mM of dNTPs mix (invitrogen), 2 mM of Mgcl_2_, and 0.05 U/μL Taq DNA polymerase (invitrogen). PCR amplifications were performed in an automated thermal cycler (Biometra Co., Germany). Table 1 summarized the primer sequences and the expected size of PCR products. The following cycle conditions were used: for *vacA*: 35 cycles of 1 min at 94°C, 1 min at 53°C, and 1 min at 72°C; for *cagA*: 1 min at 94°C, 1 min at 56°C, and 1 min at 72°C; for *iceA*: 1 min at 94°C, 1 min at 56°C, and 1 min at 72°C, and for *oipA*: 1 min at 94°C, 1 min at 56°C and 1 min at 72°C. All runs included one negative DNA control consisting of PCR-grade water and two or more positive controls (26695, J99, SS1, Tx30, 88-23 and 84-183). The amplified PCR products were resolved in 1.5% agarose gels stained with ethidium bromide and visualized under a short wave length ultraviolet light source.

**Table 1:** PCR primers for amplification of *cagA*, *vacA*, *iceA *and *oipA *sequences

Region	Primer	Primer sequence (5'-3')	Size of PCR product (bp)	References
*cagA*	F1	GATAACAGCCAAGCTTTTGAGG	349	[36]
	B1	CTGCAAAAGATTGTTTGGCAGA		
*vacA*				
m1	VA3-F	GGTCAAAATGCGGTCATGG	290	
	VA3-R	CCATTGGTACCTGTAGAAAC		
m2	VA4-F	GGAGCCCCAGGAAACATTG	352	
	VA4-R	CATAACTAGCGCCTTGCAC		
s1/s2	VA1-F	ATGGAAATACAACAAACACAC	259/286	[12]
	VA1-R	CTGCTTGAATGCGCCAAAC		
s1b	SS3-R	AGCGCCATACCGCAAGAG	187	
	VA1-R	CTGCTTGAATGCGCCAAAC		
s1a	SS1-R	GTCAGCATCACACCGCAAC	190	
	VA1-R	CTGCTTGAATGCGCCAAAC		
s2	SS2-R	GCTAACACGCCAAATGATGC	199	
	VA1-R	CTGCTTGAATGCGCCAAAC		
*iceA*1	iceA1-F	GTGTTTTTAACCAAAGTATC	247	[37]
	iceA1-R	CTATAGCCASTYTCTTTGCA		
*iceA*2	iceA2-F	GTTGGGTATATCACAATTTAT	229/334	[37]
	iceA2-R	TTRCCCTATTTTCTAGTAGGT		
*oipA*	HPO638F	GTTTTTGATGCATGGGATTT	401	[38]
	HPO638R	GTGCATCTCTTATGGCTTT		

### Statistical analysis

Data were analyzed using *X*^2 ^test. A *p *value of < 0.05 was considered to be statistically significant.

## Results

### *cagA *genotyping

The 349-bp PCR product indicating the presence of the *cagA *gene was obtained with 173 isolates (61.6%) and 108 (38.4%) were negative. We showed *cagA *genotype discordance between antrum and fundus in 27 isolates (9.6%) (Table 2).

**Table 2:** Prevalence of *H. pylori *genotypes detected in strains

Genotype	Prevalence
*cagA*	
*cagA *+	173 (61.6%)
*cagA *-	108 (38.4%)
d(A/F)*	27(9.6%)
*vacA*	
m1s1a	5 (1.8%)
m1s1b	25 (8.9%)
m2s1a	10 (3.5%)
m2s1b	25 (8.9%)
m2s2	128 (45.6%)
m1m2	88 (31.4%)
m1s2	0 (0%)
d(A/F)*	13 (4.6%)
*iceA*	
*iceA*1	169 (60.2%)
*iceA*2	45(16%)
*iceA*1/*iceA*2	67(23.8%)
d(A/F)*	25(8.9%)
*oipA*	
*oipA *+	255 (90.8%)
*oipA *-	26 (9.2%)
d(A/F)*	24(8.5%)

d(A/F)*: discordance between strains isolated from the antrum and the fundus in the same patient

### *vacA *genotyping

The *vacA *s- and m- region genotype were determined in all strains studied. In the m-region, 88 strains contained both m1 and m2 alleles. In the strains containing one single *vacA *m allele, the m1 allele was found in 30 isolates (10.7%) and m2 in 163 one (58%).

For the s-region, in strains where a single *vacA *s allele was found, the majority 128 (45.6%) contained the s2 allele. In 65 isolates contained s1 allele (23.2%), 50 (77%) were subtype s1b while only 15 (23%) were subtype s1a.

Considering strains with only one single *vacA *genotype, and taking *vacA *s- and m-region together, three genotypes were found: s1/m1 (10.7%), s1/m2 (12.5%) and s2m2 (45.6%). The s2m1 genotype was not found in our study.

The discordance between strains taken from antrum and those from fundus was found in 13 cases (4.6%) (Table 2).

### *iceA *genotyping

**Figure 1:**
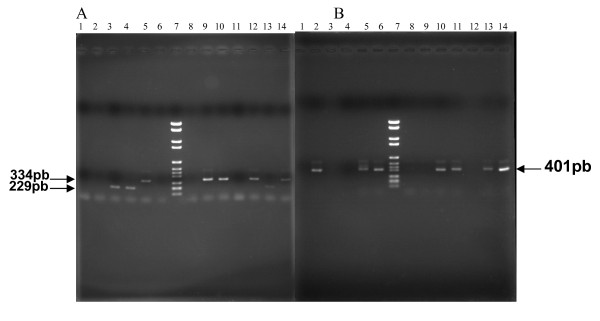
**Genotyping of *iceA2 *and *oipA *genes by PCR**. **A**: example of *iceA*2 genotyping. Lane 1, negative control without DNA, Lanes 2, 6, 8 and11, *iceA*2 negative *H. pylori *strain, Lanes 3, 4 and 13, *iceA*2 positive *H. pylori *strain (229-bp), Lanes, 5, 9, 10, 12 and 14, *iceA*2 positive strain (334-bp), Lane 7,100-bp DNA marker. **B: **example of *oipA *genotyping. Lane 1, negative control without DNA, Lanes 2, 5, 6, 10, 11, 13 and 14, *oipA *positive strains, Lane 7, 100-bp DNA marker, Lanes 3, 4, 8, 9 and 12, *oipA *negative strains.

Overall, *iceA*1 was detected in 169 strains (60.2%) of all 281 isolates and *iceA*2 was found in 45 strains (16%). In the present study, the *iceA*2 amplification yielded both the 229 bp and 334 bp fragments (figure 1), this difference in the fragment size is due to the presence of a 105 bp in - frame amplicon present in the 334 bp fragment that is absent in the 229 bp fragment [22]. Sixty-seven strains (23.8%) were positive for both *iceA*1 and *iceA*2, and discordance between isolates from antrum and fundus was seen in 25 strains (8.9%) (Table 2).

### *oipA *genotyping

The *oipA *genotype was detected in 255 (90.8%) from 281 strains, and only 26 strains (9.2%) were *oipA *negative. The discordance between strains obtained from antrum and fundus was determined in 24 strains (8.5%) (Table 2). The 401-bp PCR product indicating the presence of the *oipA *gene was shown in figure 1.

### Combined *cagA*, *vacA*, *iceA *and *oipA *genotypes

Based on analysis of the *cagA *gene (positive and negative), the *vacA *s-region (s1 and s2), the *iceA *allelic type (*iceA*1 and *iceA*2) and the *oipA *gene (positive and negative), ten different genotypic combinations were recognized (Table 3). The prevalence of each of these genotypes among the 131 strains with a single combined genotype was shown in table 3. The most prevalent genotypes were s2/*cagA*+/*iceA*1/*oipA*+ (27.5%), s2/*cagA*-/*iceA*1/*oipA*+ (23.6%), s2/*cagA*+/*iceA*2/*oipA*+ (11.4%) and s1/*cagA*+/*iceA*1/*oipA*+ (9.9%). Among the strains isolated from 24 ulcer patients (gastric and duodenal ulcer) with a single genotype, 8 strains (33.3%) contained the *vacA *s1/*cagA*+/*iceA*1 genotype while only one (4.1%) contained *vacA *s1/*cagA*-/*iceA*2. If we consider only *vacA *and *cagA *genotypes, 14 strains (58.3%) isolated from 24 ulcer patients had genotype *vacA *s1/*cagA*+, and none (0%) had *vacA *s2/*cagA*-. If we consider only *vacA *and *iceA*, 18 strains (75%) contained genotype *vacA *s1/*iceA*1, and none (0%) had *vacA *s2/*iceA*2. When we take only *vacA *and *oipA *genotypes, 21 strains (87.5%) had genotype *vacA *s1/*oipA*+ and none (0%) had *vacA *s2/*oipA*-. Comparing with strains isolated from patients having gastritis, we found that the prevalence of genotypes *vacA *s1/*cagA*+, *vacA *s1/*iceA*1 and *vacA *s1/*oipA*+ was 11.2%, 10.3%, and 16.8%, respectively. The genotype *vacA *s1/*cagA*+/*iceA1*/*oipA*+ was found in 13 (9.9%) of 131 isolates, and 8 (61.5%) of these are associated with a clinical diagnosis of peptic ulcer disease, whereas genotypes harbouring the s2 allele of *vacA *gene were predominant in the strains isolated from patients with gastritis.

**Table 3:** Combined *vacA*, *cagA*, *iceA *and *oipA *genotypes

Combination	DU*	GU*	G*	Total
s1/*cagA*+/*iceA1*/*oipA*+	4(20%)	4(100%)	7(6.5%)	13(9.9%)
s1/*cagA*+/*iceA2*/*oipA*+	4(20%)	0(0%)	4(3.7%)	8(6.1%)
s1/*cagA*-/*iceA1*/*oipA*+	8(40%)	0(0%)	3(2.8%)	11(8.4%)
s1/*cagA*-/*iceA2*/*oipA*+	1(5%)	0(0%)	4(3.7%)	5(3.8%)
s1/*cagA*+/*iceA1*/*oipA*-	2(10%)	0(0%)	1(0.9%)	3(2.3%)
s2/*cagA*+/*iceA1*/*oipA*+	1(5%)	0(0%)	35(32.7%)	36(27.5%)
s2/*cagA*+/*iceA2*/*oipA*+	0(0%)	0(0%)	15(14%)	15(11.4%)
s2/*cagA*-/*iceA1*/*oipA*+	0(0%)	0(0%)	31(29%)	31(23.6%)
s2/*cagA*-/*iceA2*/*oipA*+	0(0%)	0(0%)	4(3.7%)	4(3%)
s2/*cagA*-/*iceA2*/*oipA*-	0(0%)	0(0%)	5(4.6%)	5(3.8%)

Total	20	4	107	131

DU*: duodenal ulcer; GU*: gastric ulcer; G*: gastritis

In our study, first, we have studied the relationship between virulence genes (*vacA*, *cagA*, *iceA*, *oipA*) and the different gastroduodenal diseases, between the two groups (patients with peptic ulceration/patients with gastritis). When considering the *vacA *and *oipA *genes, the difference was statistically significant between the two groups (p < 0.001), but, when considering the *cagA *and *iceA *genes, this relation is not statistically significant (p > 0.05) (Tables 4). Second, we were interesting in studying the relationship between: a/the age of patients and virulence factors (Table 5) and b/the sex and virulence factors (Table 6). Our study showed that, among age, the difference was statistically significant between adults and children with the *cagA *and *iceA *genes (p < 0.05), but not significant when considering the *vacA *and *oipA *genes. When about sex, we have demonstrated that the difference between men and women was statistically significant among *vacA*, *oipA *and *iceA *genes (p < 0.05), which was not statistically significant with the *cagA *gene.

**Table 4:** *cagA*, *vacA*, *iceA *and *oipA *status of *H. pylori *strains with different gastric diseases: a comparison between patients with peptic ulceration, and with gastritis

	PU*	G*	*p*
cagA+	53 (67.9%)	114 (58.4%)	> 0.05
oipA+	63 (80.7%)	186 (95.3%)	< 0.001
iceA1	51 (65.3%)	113 (57.9%)	> 0.05
iceA2	12 (15.3%)	33 (16.9%)	
iceA1/A2	15 (19.2%)	49 (25.1%)	
m1s1	12 (15.3%)	18 (9.2%)	< 0.001
m2s1	17 (21.8%)	17 (8.7%)	
m1m2	49 (62.8%)	32 (16.4%)	
m2s2	0 (0%)	128 (65.6%)	

PU*: peptic ulceration; G*: gastritis

**Table 5:** Relation between the age of patients and virulence factors:

	Adults	Children	*p*
*cagA *+	155	18	< 0.001
*oipA*+	209	46	> 0.05
*iceA*1	137	32	0.05
*iceA*2	43	2	
iceA1/iceA2	53	14	
m1s1	26	4	> 0.05
m2s1	31	4	
m1m2	72	16	
m2s2	104	24	

**Table 6:** Relation between the sex of patients and virulence factors:

	Male	Female	*p*
*cagA *+	84	89	> 0.05
*oipA*+	111	144	< 0.05
*iceA*1	78	91	
*iceA*2	17	28	0.05
iceA1/iceA2	40	27	
m1s1	19	11	
m2s1	19	16	< 0.001
m1/m2	49	39	
m2s2	40	88	

## Discussion

The predominant genotype in strains that were positive for *H. pylori *by PCR was the *oipA *(90.8%), followed by the *cagA *gene (61.6%), and the *iceA*1 gene (60.2%), while the *iceA*2 was amplified only in 45 strains (16%). Our results were in agreement with other studies conducted in Europe, Central and South America, and East Asia where a higher prevalence (67% or more) of the *cagA *genotype was reported [22]. In Turkey [23], Korea and Japan, the prevalence of the *iceA*1 genotype in patient with duodenal ulcer were respectively 68.8%, 69.8% and 62.5% which is similar to our study, whereas *iceA*2 was prevalent in the USA and Columbia [24]. The already mentioned similitude can be defined by the epidemiological resemblance in these countries.

For the *vacA *genotype, and when considering a single combined genotype, our results showed that the *vacA *s2 allele was predominant (45.6%) followed by the *vacA *s1 allele (23.2%). A study in Kuwait reported that *vacA *s1 and s2 types were detected in approximately equal numbers in biopsies obtained from patients of Middle-Eastern origin, while North Africans were predominantly infected with the s2 type [25].

The prevalence of *cagA*+ strains in Tunisian peptic ulcer and those with gastritis was similar to that shown in the study from South Africa [26].

In a previous report by van Doorn *et al *(19), an association was found between the *iceA*1 allele and peptic ulcer disease. Other studies from Asia were suggesting that *vacA*, *cagA *and *iceA *genotypes were not associated with peptic ulcer disease [27]. These findings may reflect important geographic differences between *H. pylori *strains and patients. As reported by van Doorn *et al*, *H. pylori *genotypes are not uniformly distributed over the world [22]. When considering the *vacA *and *oipA *genes, our results showed that there is a significant difference between the two studied groups (p < 0.001), but, *cagA *and *iceA *genes were not associated with gastroduodenal diseases in our study (p > 0.05). The *oipA *(HpO638) outer membrane protein expression was linked to severe inflammation and the induction of IL-8 secretion. In one Turkish study, *oipA *(HpO638) gene was highly associated with peptic ulcer disease (92.9%) than with gastritis (80.9%) [28]. In other studies, no difference in prevalence of *cagA *genotypes was found between peptic ulcers and other gastroduodenal diseases [29]. Yamaoka *et al *[24] reported that there was no association between the *iceA*, *vacA *or *cagA *status and clinical outcome in studied patients.

In our study, the *oipA *gene was found in 100% of gastric ulcer. Several studies failed to show a relationship between the gene status and clinical symptoms in several patient populations, this might be due to that patient selection is extremely important and the study group should be sufficiently large and diverse with respect to genotypes and clinical symptoms.

As for age, we have demonstrated that the difference between children and adults was statistically significant about the *cagA *and *iceA *genes, but not with the *vacA *and *oipA *genes. Regarding the distribution of gene virulent factors according to sex, no difference seen in term of *cagA *gene, but the difference was statistically significant among the *vacA*, *iceA *and *oipA *genes. In other reported studies, there were no significant differences in frequencies of *H. pylori *virulence-associated genes, between children and adults, and the strains from men exhibited genotypes similar to those in women [30]. Laila FN *et al *[31] have concluded in their study that the presence of certain genotypes was not significantly associated with the age or gender of the patient.

The detection of multiple genotypes implies the presence of multiple strains in a clinical sample. Considering *vacA *and *iceA *genes in our study, the presence of multiple *H. pylori *strains in a single biopsy specimen was found respectively in 31.4% and 23.8%. Then, when comparing the genetic differences in isolates of *H. pylori *from the antrum and the fundus, we determined that patients were infected with two or more strains of different *cagA*, *vacA*, *iceA *and *oipA*, and the percentage of discordance was respectively in 9.6%, 4.6%, 8.9% and 8.5%. Consequently, if multiple genotypes are found, this is a strong indication for the presence of multiple strains. It may be speculated that more than one strain may be acquired in childhood, especially in countries with a very high prevalence of *H. pylori*, but, it is not known whether multiple strains colonize simultaneously (coinfection) or at different time points (superinfection), and several studies demonstrated that coinfection or superinfection are common [32,33]. The co-existence of more than one strain in the same individual may reflect the capacity of *H. pylori *to evolve genetic variations during the long-term colonization from childhood [34], and the dynamics of co-colonization by multiple strains has been studied in animal models [35]. Since the prevalence of multiple strains colonization is clinically important in our study, there is a clear need to be considered when planning therapeutic strategies to avoid the risk of the emergence of such strains.

The present study showed that patients with peptic ulcer disease and those with gastritis were almost infected similarly by multiple strains of *H. pylori*. This underlines that the positive associations between *H. pylori *genotypes and peptic ulcer disease do not imply that patients without ulcer disease cannot be infected with high-risk *H. pylori *genotypes.

The combination of the distinct *vacA*, *cagA*, *iceA *and *oipA *genotypes illustrated the mosaic composition of the *H. pylori *genome. Strains typed as *vacA *s2/*cagA*+/*iceA*+/*oipA*+ were more prevalent than those typed as *vacA *s1/*cagA*+/*iceA*+/*oipA*+ and which are associated to severe pathologies.

In conclusion, we have examined the prevalence of *vacA*, *cagA*, *iceA *and *oipA *genotypes of *H. pylori *strains clinically isolated in Tunisia. No significant correlation was found between the expression of *cagA *and *iceA *genes and the two groups of studied patients, but the difference was statistically significant with the *vacA *and *oipA *genes. As for age, the difference between children and adults was statistically significant with the *cagA *and *iceA *genes, but not with *oipA *and *vacA *genes. Regarding the distribution of virulence genes to sex, the difference was statistically significant among *oipA*, *iceA *and *vacA *genes, but not with *cagA *gene.

In Tunisia, it is so possible that the high prevalence of infection with virulent factors contributes to the characteristics of *H. pylori *infection, but not used to discern the risk of developing serious gastroduodenal diseases in the host. Our results showed that Tunisian patients were colonized by one or more strains of *H. pylori *in relation of presence of *vacA *m1/m2 and *iceA*1/*iceA*2 in the same biopsy, than, the discordance between strains isolated from antrum and fundus was higher, and it is in favour of multicolonization.

## Competing interests

The authors declare that they have no competing interests.

This prospective multicentre study was performed in Microbiology laboratory-Rabta University Hospital, Tunis, Tunisia in research unit UR04SP08.

## Authors' contributions

BMK was responsible for the collection of biopsies, *H. pylori *conventional culture, had carried out the molecular genetic studies, performed the statistical analysis, drafted the manuscript and participated in the design of the study. ZM and MA were participated in the design of the study. FA, BMN, NT, MA and ST were helped to the constitution of *H. pylori *strains collection. FC had conceived of the study, participated in its design and coordination and helped to draft the manuscript. Finally, BC had helped to molecular genetic studies and to draft the manuscript.

All authors have read and approved the final manuscript.
